# Squamous cell carcinomas of the lung and of the head and neck: new insights on molecular characterization

**DOI:** 10.18632/oncotarget.7732

**Published:** 2016-02-25

**Authors:** Valentina Polo, Giulia Pasello, Stefano Frega, Adolfo Favaretto, Haralabos Koussis, Pierfranco Conte, Laura Bonanno

**Affiliations:** ^1^ Medical Oncology 2, Istituto Oncologico Veneto IRCCS, Padova, Italy; ^2^ Department of Surgery, Oncology and Gastroenterology, Università degli Studi di Padova, Padova, Italy

**Keywords:** squamous cell carcinoma, lung, head and neck, smoke, HPV

## Abstract

Squamous cell carcinomas of the lung and of the head and neck district share strong association with smoking habits and are characterized by smoke-related genetic alterations. Driver mutations have been identified in small percentage of lung squamous cell carcinoma. In parallel, squamous head and neck tumors are classified according to the HPV positivity, thus identifying two different clinical and molecular subgroups of disease.

This review depicts different molecular portraits and potential clinical application in the field of targeted therapy, immunotherapy and chemotherapy personalization.

## INTRODUCTION

Squamous cell carcinoma of the lung (LSCC) is the second most common histological subtype of non-small cell lung cancer (NSCLC) having smoking habit as the most recognized risk factor [1, 2].

LSCC currently represents 30% of lung cancers in men and 20% in women [3]. Its incidence has been decreasing worldwide since 1979 even though an apparent increase in LSCC incidence has been recently registered, probably as a consequence of a widespread use of immunohistochemistry for classifying lung cancer histotypes and the subsequent decrease in not otherwise specified NSCLC diagnosis [4]. Squamous cell carcinoma (SCC) is the most frequent histotype among the head and neck tumors. In the latest ten years a substantial global variation in epidemiology of head and neck squamous cell carcinoma (HNSCC) has been observed: laryngeal and oral SCC incidences are strongly associated with changes in smoking habit, while oropharyngeal cancers are increasing especially in young people and in some European and Asian nations mainly due to human papilloma virus (HPV) infection [5, 6]. Many studies have also detected HPV in NSCLC, ranging widely from 0 to 78%, thus suggesting a potential carcinogenic role of HPV infection in at least a subset of lung cancer cases. However, recent comprehensive analyses reported that the variability in HPV detection rates in lung cancer (especially adenocarcinoma and LSCC) could be a function of the geographical origin of the studies. HPV detection was more prevalent in Asia and South America compared to Europe, Australia, and North America [7, 8]. A recent study by Chang *et al*. supports this finding showing no HPV detection in a North American population of NSCLC [9].

LSCC and HNSCC may present as synchronous or metachronous tumors, also in relationship with common pathogenesis [10]. They also share morphological microscopic features, making differential diagnosis on metastatic sites highly challenging. Currently, the differentiation between distant metastasis and second primary tumors is made on the basis of clinical criteria. Molecular characterization could be useful in this setting, as shown in a small study in which loss of heterozygosity and *TP53* mutation analysis have been successfully used for differential diagnosis [11].

Molecular characterization is key in order to develop targeted therapies for solid tumors. Recently, wide genotyping of SCC of the lung and the head and neck has been performed, highlighting specific molecular traits. As far as head and neck is concerned, a different genetic profile according to HPV infection has been depicted. The molecular characterization of these diseases could potentially open new perspectives of tailored treatments for specific subsets of patients.

## COMMON MOLECULAR ALTERATIONS IN SMOKE-RELATED SQUAMOUS CELL CARCINOMAS

### Genetic alterations and common pathogenesis

LSCC and HNSCC are clinically and genetically heterogeneous diseases, but they share a number of molecular characteristics suggesting similar biology and pathogenesis.

**Figure 1:**
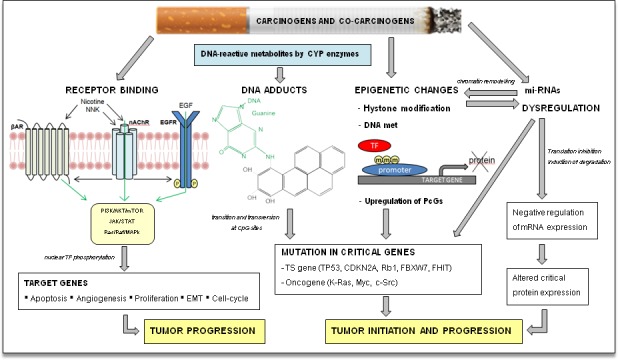
Tobacco smoke carcinogenesis The main recognized mechanism of tobacco initiation of carcinogenesis is the formation of DNA adducts by polycyclic aromatic hydrocarbons and aromatic amines, with consequent deregulation of critical oncogenes and/or tumor suppressor (TS) genes. Tobacco smoke can influence protein expression of squamous cell cancer patients through induction of reversible epigenetic changes and miRNAs dysregulation; these pathways are also inter-related. Nicotine and its oncogenic derivatives are unable to initiate carcinogenesis, but can promote tumor growth through activation of multiple kinase cascade, acting directly on nicotinic acetylcholine receptor (nAChR) or indirectly on parallel cell surface receptors. βAR, beta adrenergic receptor kinase; CDKN2A, cyclin-dependent kinase inhibitor 2A; CpG, C-phosphate-G; c-Src, cellular Src kinase; CYP, cytochrome p450; FBXW7, F-box and WD repeat domain containing 7; FHIT, fragile histidine triad; m, methyl-CpG; Myc, myelocytomatosis oncogene; nAchR, Nicotinic acetylcholine receptor; NNK, nicotine-derived nitrosamine ketone; PcGs, polycomb-group protein; Rb1, retinoblastoma1; TF, transcription factor.

The two diseases recognize smoke as a major risk factor, thus implying common elements both in pathogenesis and in the pattern of genetic alterations. Tobacco-related carcinogenesis is a typical model of complex multi-step carcinogenesis characterized by larger numbers and complexity of DNA alterations in these two tumors compared to other solid malignancies. Tobacco smoke is implicated in LSCC and HNSCC pathogenesis in different ways. In addition to the well-known mechanism of DNA adducts induction, several other mechanisms have been described, with a relevant role also for epigenetic alterations and micro-RNA deregulation (Figure 1). More than sixty known carcinogens have been detected in cigarette smoke; among these, tobacco-specific N-nitrosamines, polycyclic aromatic hydrocarbons, and aromatic amines are currently recognized as the strongest tumorigenic substances [12]. The majority of the smoke-related carcinogens require metabolic activation to react with DNA and cause DNA adducts formation (Figure 1). Transitions and transversions at CpG sites are typically found in genes commonly altered in smoking patients, such as *KRAS, TP53* and *RB* [13–15]. Interestingly, the pattern of mutations in HNSCC is different according to HPV positivity. HPV-negative tumors, where carcinogenesis is mainly smoke-related, show transversions at CpG sites more frequently than HPV-positive tumors, known to be led by virus-mediated carcinogenesis [12]. On the contrary, mutations at Tp*Cp sites are more common in the latter group. More recent studies demonstrated that nicotine and its oncogenic derivatives are unable to initiate tumorigenesis, but they promote the subsequent steps of carcinogenesis: tumor growth, cell proliferation, migration, invasion, evasion of apoptosis, epithelial-to-mesenchymal transition, tumor angiogenesis and immune-response down-regulation (Figure 1). These effects are mediated especially by the binding to the nicotinic acetylcholine receptors (nAChR) and subsequent activation of multiple signaling cascades such as *JAK/STAT, Ras/Raf/MAPK*, and *PI3K/AKT*. In addition, nicotine and nitrosamines have been shown to induce cell-cycle progression, by the overexpression of cyclins and decreased levels of cyclin-dependent kinase (Cdk) inhibitors. These mechanisms mediated by tobacco components on already initiated tumors could also explain the increased probability of resistance to chemotherapy and radiotherapy reported in patients still smoking during cancer treatment [16].

The major genomic determinants related to smoke exposure concern mainly tumor suppressor genes, including *TP53, CDKN2A* and *RB1* [13]. In particular, about 85% of smoke-related LSCC and HNSCC present loss of function in the tumor suppressor gene *TP53* while loss of function of *CDKN2A* is found in about 20% of cases [12, 17]. A large amount of alterations affects genes directly involved in squamous cell differentiation implicating their dysregulation as major driver of SCC carcinogenesis. Typical molecular features of squamous cell differentiation are loss of chromosome 3p and gain of chromosome 3q [18–20]. Also inactivating mutations of the *NOTCH* family members genes have been associated with squamous differentiation [21]. In addition, LSCC and HNSCC are characterized by frequent alterations of oncogenic pathways typically involved in many solid tumors such as the *PI3K/AKT*, the *NFKB1* and the Hippo pathway [12, 22–24]. Multiplatform genomic and proteomic analysis confirmed that LSCC and HNSCC share common molecular features and in particular they were classified as a unique subtype together with a subset of bladder cancer, another solid tumor typically related to smoke exposure [25]. A more recent work identified a set of 8 genes altered with significantly different frequencies in SCC originating from different organs compared to non-SCC, suggesting the existence of a “squamousness” signature [26]. The Cancer Genome Atlas (TCGA) published the results of the comprehensive multi-platform genomic characterization of a large cohort of LSCC and HNSCC in 2012 and 2015, respectively. These data unveiled main molecular alterations in the two diseases and provided further compelling evidence of the similarity of the two neoplasms in terms of genetic and epigenetic landscape [12, 17].

### Molecular subtypes classification

**Figure 2:**
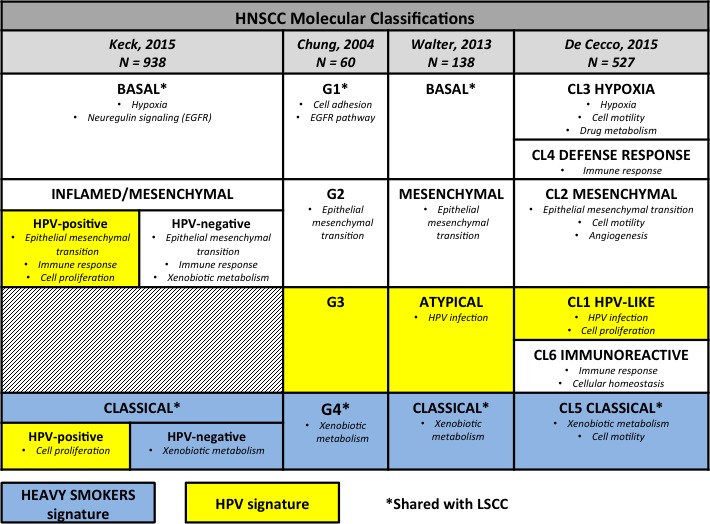
Molecular subtypes in head and neck squamous cell carcinomas The figure summarizes the molecular subtypes of head and neck squamous cell carcinomas according to the four different molecular classifications available in the literature with the main dysregulated functional pathways for each subgroup. We highlighted molecular subtypes associated with human papillomavirus infection by yellow boxes, those associated with smoking habits by blue boxes, and we marked by * the subgroups corresponding to lung squamous cell carcinoma expression subtypes. HNSCC, head and neck squamous cell carcinoma; HPV human papillomavirus; LSCC, lung squamous cell carcinoma.

Based on genomic characterization, different molecular subtypes of LSCC and HNSCC have been defined and validated by the evaluation of the dominant gene expression pattern (Figure 2). The milestone of this kind of study is that expression subtypes should be reproducible from a statistical and biological point of view, associated with different clinical pattern and corresponding to different cellular type with different biological behavior, thus paving the way to further studies on different pathogenesis and therapeutic approach. The four mRNA expression subtypes of LSCC are named classical, basal, secretory, and primitive [27], while four different classifications have been proposed for HNSCC [28–31] (Figure 2).

This review focuses on the molecular subtypes associated with smoke exposure and, in the paragraph below, on those related to HPV infection.

In both malignancies and according to all classification of HNSCC, the classical subtype presents the highest percentage of current smokers and it does not show appreciable similarity to any specific normal cell type. This molecular subgroup of SCC is characterized by the highest overall methylation and chromosomal instability, as well as overexpression of genes involved in xenobiotic metabolism, indicating a sustained response to cigarette smoke. Among HNSCC, the classical subtype occurs especially in laryngeal tumors, consistently with the correlation with tobacco exposure [12].

Moreover, broad amplification of chromosome 3q, widely recognized as associated with SCC, particularly defines the classical subtype and results in the overexpression of three oncogenes: *SOX2, TP63* and *PIK3CA* [32]. *SOX2* gene is a key transcription factor that promotes normal squamous differentiation and it is overexpressed in about 20% of LSCC. In SCC, it works as a lineage-survival oncogene, probably even activating additional pathways controlled in early pluripotent cells [33]. The transcription factor *TP63* gene is expressed as multiple isoforms with different functions, including the truncated amino-deleted isoform and the full length one. The short deltaN isoform (also called p40) is known to be the most commonly expressed isoform in squamous tumors, and it functions as an oncogene, promoting growth and survival by competing for p53 binding sites [20]. As a matter of fact, p63 overexpression, evaluated by immunohistochemistry, is one of the main markers used by the pathologists to define squamous cell differentiation in addition to specific morphology [34]. The *PIK3CA* gene is a key regulator of cell growth, metabolism, and survival and it is responsible for PI3K activity and phosphorylated Akt expression. *PIK3CA* activating mutations and copy number gains occur independently of each other, and either molecular event may be sufficient to drive the cell towards tumorigenesis by promoting cell growth and invasion [35, 36]. In SCC, alterations of the *NFR2-KEAP1*/*CUL3* pathway are also frequently reported. In different studies about LSCC and HNSCC, mutations and copy number alterations of *NRF2* and *KEAP1* and/or deletion or mutation of *CUL3* have been detected, especially in the classical subtype [12, 17, 37]. *NRF2* and *KEAP1/CUL3* encode for proteins that bind to each other and have been shown to promote the cell response to oxidative damage. Indeed, the transcription factor Nrf2 mediates gene induction of numerous cytoprotective enzymes in response to environmental and endogenously derived oxidative/electrophilic agents. On the other hand, under physiological conditions, low cellular concentrations of Nrf2 are maintained by proteasomal degradation through a Cullin3-Keap1 system. Hence, mutations in *KEAP1* and *CUL3* are mutually exclusive with mutations in *NRF2*, and each of these leads to a constitutive activation of Nrf2, promoting cancer cell survival [38–40].

Overall, the classical subtype is enriched in genes involved in regulation of oxidant stress and glutathione metabolism, xenobiotic metabolism, and secretion, such as *GPX2, ALDH3A1, AKR1C1/3, TXNRD1,* and *GSTM3* [27, 29, 30].

Even though the classical subtype can be clearly associated with smoke exposure and genetic alterations associated with tobacco-related damage, the presence of different patterns of genetic alterations does not exclude smoke exposure. On the contrary, also in the other molecular subtypes most of the patients were former smokers, although exhibiting molecular alterations in different pathways [28–31]. Figure 2 shows the characteristics of molecular subtypes in HNSCC according to different classifications, highlighting the subtypes corresponding to the LSCC classification.

## SQUAMOUS HEAD AND NECK CANCERS IN HPV-POSITIVE PATIENTS: A NEW MOLECULAR ENTITY

Although HNSCC is widely viewed as comprised of two distinct clinical and biological entities, the genetic landscape of HPV-positive tumors remains unclear since data from large HPV-positive cohorts are still missing. The first study on molecular alterations in HNSCC including a large proportion of HPV-positive tumors (*N* = 51 samples, 42.5% of cases) has been published in 2015 [37]. This comprehensive genomic analysis confirmed the hypothesis of distinct mutational and copy-number profiles between HPV-positive and HPV-negative tumors, as suggested by previous studies on small HPV-positive series [12, 41, 42]. Some critical issues should be considered in discussing the results. First, a considerable proportion of HPV-positive patients reported significant smoking history. In addition, HPV-positive status confers a favorable prognosis, but also smoke exposure is independently associated with survival and a significantly increased risk of death is associated with each additional pack-year of tobacco smoke. The effect of tobacco on clinical outcome is similar among patients with HPV-positive tumor (hazard ratio, 1.01; 95% CI, 1.00 to 1.02) and HPV-negative cases (hazard ratio, 1.01; 95% CI: 1.0-1.03) [43]. This finding is likely to be related to the four-to-six-fold higher mutational burden detected in such HPV-positive patients compared to HPV-positive tumors without smoking history. These additional genetic alterations are linked to tobacco-related carcinogens, as confirmed by the presence of *KRAS* and *TP53* mutations. Thus, HPV-positive tumors in smoking patients and HPV-negative tumors share molecular alterations such as 3q amplification, PI3K signaling [44], *NOTCH* aberrations [45] and SMAD signaling. However, HPV-positive tumors are more enriched in mutations/copy number variations in oncogenes compared to HPV-negative patients [46].

HPV infection is implicated in HNSCC tumorigenesis through different mechanisms. The best known element is the overexpression of viral E6-E7 proteins that act as oncoproteins promoting p53 or Rb degradation, activation of hTERT, neutralization of the inhibitory effect by CDK inhibitors on cell cycle; thus, HPV infection activates the process of carcinogenesis by deregulating fundamental cellular pathways [47]. Moreover, beyond the impairment of immune surveillance in order to promote viral persistence in HPV-infected cells, different immune evasion strategies are involved in HPV-driven tumors including the recruitment of immune cells with immunosuppressive properties [48].

HPV DNA is present in tumor cells both as integrated into the human genome and as episome, nonintegrated into the genome. A recent study evaluated the effect on carcinogenesis of the integration of the viral genome in 25 cases of HPV-positive HNSCC. The data suggest that, beyond the expression of viral oncoproteins E6 and E7, HPV promotes oncogenesis by the alteration of host genome at the sites of integration. The effects of viral DNA integration into human genome include disruption of tumor suppressor genes, oncogene amplifications, interchromosomal rearrangements and altered methylation. Examples of genes altered by viral DNA integration are *RAD51B*, leading to loss of DNA-repair function, and *NR4A2*, a nuclear transcription factor which acts as an oncogene, especially by inhibiting apoptosis [49].

Recently Gaykalova *et al*. confirmed a different underlying biology of HPV-positive and HPV-negative tumors by evaluating the activity of key transcription factors; their data suggested that HPV-positive and HPV-negative tumors show specifically different patterns of alterations in transcription factor pathways, including *AP1, STATs, NF-κB* and *p53* [50].

The most commonly altered pathway in HPV-positive tumors is *PI3K/AKT* that is involved in regulating the signaling of different processes such as apoptosis, metabolism, cell proliferation, and cell growth. Mutations and/or copy number variations of the catalytic domain (PIK3CA) of PI3K are present in about 20-30% of cases, while, more rarely, mutations occur in other genes of the same pathway: *PTEN, AKT1, PIK3R1, TSC1,* and *TSC2*, thus making the pathway a potential therapeutic target. Other potentially druggable alterations of particular interest are *FGFR2/3* mutations occurring in about 25% of HPV-positive cases, while in HPV-negative patients *FGFR1* amplifications are more common [37].

Interestingly HPV-positive tumors, especially in never/light-smoker patients, are enriched also in alteration to DNA damage repair pathway including the genes *BRCA1, ATM, BRCA1, BRCA2, FANCG, FANCA*, and *FANCD2* [37]. We can speculate that inefficient DNA repair capacity in these tumors may in part explain the increased sensitivity to chemotherapy and platinum-based treatment, specifically. Specific data on this issue are missing, anyway.

Moreover, wide genome analyses highlighted the higher presence of genetic alterations in immune-related genes (*HLA-A* and *HLA-B*) and this point may be directly related to viral tumorigenesis [12, 37].

Finally, two genes are exclusively altered in HPV-positive HNSCC: *DDX3X*, involved in RNA processing, and CYLD, a multifunctional deubiquitinase involved in negative regulation of NF-κB signaling [37]. In particular, CYLD correlates also with HPV-related cervical cancer, thus suggesting the idea of a specific role in virus-related carcinogenesis. In preclinical models, under hypoxic conditions, the HPV-encoded E6 protein promotes inactivation and degradation of the CYLD tumor suppressor, resulting in prolonged activation of NF-κB pathway [51].

In previous molecular classification HPV-related tumors have been mainly included in the atypical subgroup (Figure 2); however the overall small number of HPV-positive patients included in the analysis affected the final classification, probably impairing the identification of specific features in the HPV-related subgroup. The more recent molecular nomenclature of HNSCC by Keck *et al*. included also a large cohort of HPV-positive patients in order to provide a comprehensive overview of HPV-negative as well as HPV-positive HNSCC. According to this classification, two distinct subgroups of HPV-positive tumors fall in the classical and in the mesenchymal subtype, respectively (Figure 2). HPV-positive patients without smoking history share main molecular alterations with the HPV-negative counterpart with the exception of smoking-associated pathways. Indeed, in both subtypes around 80% of HPV-negative patients were heavy smokers *versus* 40% of HPV-positive patients. The classical subtype is characterized by the overexpression of cell-cycle genes and cell division cycle protein kinase (“proliferative signature”), while the main theme of the inflamed/mesenchymal subtype is the expression of immune response genes related to the enrichment of cytotoxic T-cell infiltration. This subgroup also shows the expression of mesenchymal genes and downregulation of epithelial differentiation markers while tumors are histologically poorly differentiated. These two HPV-related subtypes exhibit significant differences in terms of morphology and molecular pattern and, importantly, the mesenchymal subtype is characterized by a better survival compared to classical HPV-positive tumors. This finding may explain the clinical heterogeneity observed between HPV-positive patients with different response to treatment and highlights potential clinical application of molecular characterization and subclassification among HPV-positive HNSCC [30].

In the latest classification based on a meta-analysis approach combining multiple datasets, even if the HPV status was not available, one molecular cluster has been defined as HPV-like. This subgroup shows the up-regulation of genes related to HPV infection and cell proliferation with a significant enrichment in oropharyngeal cases and it is characterized by the best outcome in terms of prognosis compared to other clusters [31].

## SQUAMOUS CELL CARCINOMA OF THE LUNG IN NEVER-SMOKER PATIENTS: TO BE EXPLORED

Currently, few data are available concerning molecular profiling in LSCC of never smokers. LSCC in non-smokers is an exceptional finding and it is often supposed to be related to professional exposure and related carcinogenesis. Nevertheless, in high-volume thoracic surgery centers, LSCC cases are observed even in non-smokers without known professional exposure.

In the TCGA project, as well as in another cohort of East Asians patients [52], most patients (96%) reported a history of tobacco use and no conclusions may be drawn for non-smokers [17]. One of the few studies evaluating genomic alterations in different histological subtypes and according to smoking status has been conducted in Chinese lung cancer patients. The authors investigated a spectrum of driver genes, including *EGFR, KRAS, c-Met, PIK3CA, BRAF, STK11, PTEN, EML4-ALK* fusion gene, *DDR2*, and *FGFR2*. They found only *EGFR* (8.0%), c-Met (2.8%), and *PIK3CA* (2.6%) alterations in the non-smoker LSCC subgroup, without alterations of other genes analyzed. This finding suggests that this subgroup recognizes pathogenic mechanisms that differ from known driver gene alterations of lung adenocarcinoma and SCC of the smokers [53]. Consistently, another analysis on 185 Chinese patients confirmed the detection of *EGFR* mutations among LSCC tumors ranging from 6% to 17%, with high correlation with the absence of smoking exposure [54].

In another small series of Chinese LSCC patients, the authors evaluated targetable alterations according to gender. In this population, the percentage of smoking patients is significantly lower among females compared to males (5.3% *versus* 90%). However no significant difference in the mutational frequencies of *EGFR, KRAS, PTEN, ALK,* or *FGFR1* have been observed between males and females. Only *PIK3CA* mutations were significantly less common in females, and thus in never smokers, than in males, but the small sample of this study (38 females and 40 males) limits the interpretation of the results [55].

In conclusion, mutational landscape and consequently underlying biology in never-smoker LSCC tumors are still largely unexplored. Probably, wide genome characterization of LSCC samples from non-smoker patients could provide interesting data and potentially open new therapeutic perspectives.

## MOLECULAR CHARACTERIZATION AND THERAPEUTIC PERSPECTIVES

### Targeted therapy

In the latest years several efforts have been made to introduce genomic tests and targeted therapies in cancer treatment [56–59]. However, SCC is a deadly disease for which platinum-based chemotherapy is still the mainstay of treatment. Currently, cetuximab, an anti-EGFR monoclonal antibody, is the only approved targeted compound for the management of HNSCC [60, 61]. Regarding LSCC, erlotinib is the only targeted treatment used in clinical practice and has been studied in patient population without specific molecular characterization; two different *EGFR* antibodies and another *EGFR-HER2* tyrosine kinase inhibitor also have been investigated in phase III trials, here again in unselected patients [62–64].

The two main goals of comprehensive genomic characterization are to improve the knowledge on pathogenesis and to identify driver somatic alterations for which targeted therapies already exist or are under evaluation [65, 66]. As reported in Table 1, various targeted agents are in different stages of development on the basis of current genome-wide sequencing and copy number data.

**Table 1:** Targeted agents in clinical development (active phase II and III trials) in patients with lung squamous cell carcinoma and head and neck squamous cell carcinoma

Target	SCC	Frequency of genetic alteration	Targeted drug	Phase of development
**PIK3**	LSCC	16%	LY3023414 GDC-0032	II II/III
	HNSCC	16-56%	BKM120	II
**mTOR**	LSCC		Sirolimus LY3023414 AZD2014 (TORC 1/2) MLN0128 (TORC 1/2)	*I/II* II II II
**CDK4/6**	LSCC		Abemaciclib	II
	LSCC and HNSCC		Palbociclib isethionate	II/III II
**FGFR (Mut/Ampl)**	LSCC	2-7%	AZD4547 Dovitinib	II/III II
**FGFR (Mut/Ampl)** **-VEGFR**	LSCC	2-7%	Nintedanib Lucitanib	II *II*
	HNSCC	1-24%	Pazopanib	II
**HGF/c-MET**	LSCC		Rilotumumab Capmatinib	II/III *II*
**ALK**	LSCC		AP26113	*II*
**PARP**	LSCC		Veliparib	III
**EGFR (Mut/Ampl)**	LSCC	9%	Icotinib Nimotuzumab Necitumumab	II II I/II
	HNSCC	6-15%	Afatinib Cetuximab *plus* dasatinib Erlotinib Panitumumab	III II II II
**HER3**	LSCC	2%	MM-121	*II*
**pan-HER**	HNSCC		HM781-36B	II
**XPO1**	LSCC HNSCC		Selinexor	II
**Hsp27**	LSCC		Apatorsen	II

LSCC, lung squamous cell carcinoma; HNSCC, head and neck squamous cell carcinoma; Mut, mutation; Ampl, amplification

Currently, the PI3K pathway is one of the most promising therapeutic targets for both malignancies, on the basis of the high rate of *PIK3CA* mutations and of the presence of alterations in other genes involved in this pathway. Active clinical trials evaluating PI3K/AKT/mTOR targeted agents, including PIK3CA and mTOR inhibitors, are ongoing in both diseases; however, no significant clinical benefit has been demonstrated to date, even though strong preclinical rationale is widely recognized [67]. Other frequently altered targets in SCC are members of the FGFR family, characterized by the amplification of *FGFR1* or mutations of *FGFR2/3*, which might be targeted by specific agents in development through early phase clinical trials [68–70]. Furthermore, mutations in the *DDR2* kinase gene in about 4% of LSCC have been documented; such mutations lead to cellular transformation making this tyrosine kinase gene a promising target [71]. Although *DDR2* has not been a major focus of drug development efforts, a recent analysis showed that *ABL* kinase inhibitors such as imatinib, nilotinib, and dasatinib have activity against *DDR2* [72] and potential benefit of these agents in this setting are under investigation.

On the basis of available preclinical data, the design of biomarker-driven, multi-arm, randomized trials is likely to be a promising approach to promote the efficient development of targeted therapies, although a recent study has highlighted that the rarity of driver mutations may limit the feasibility of this approach [73]. According to this strategy, the National Cancer Institute has designed a series of clinical studies on various tumors by selection of patients through the identification of actionable molecular alterations with next-generation sequencing (NGS) technologies; statistical analysis has been planned for deeming the experimental agent an improvement over standard therapy. Regarding LSCC, patients with advanced disease progressing after first-line therapy receive their treatment according to molecular profile resulted from a NGS panel of 250 selected genes. Standard second-line chemotherapy is compared to targeted agent in the presence of potentially druggable alterations or to immunotherapy in the absence of driver mutations [74].

In the present review, rather then listing all the predictive biomarkers suitable for the evaluation of new targeted compounds in the management of HNSCC and SCC, we would like to highlight some key issues. First, as reported above, tobacco-related SCC, especially LSCC and HPV-negative HNSCC, are genetically complex malignancies and it is unlikely that most cases may benefit from targeted monotherapy. The rationale for the development of targeted therapy lay on the evidence of tumor cells addicted to one or few genes for maintenance of the malignant phenotype; on the contrary, malignancies characterized by many genetic and epigenetic alterations may potentially benefit from multiple biological agents targeting different drivers. A new potential model for the development of new targeted treatment specifically concerns tumors predominantly enriched in tumor suppressor genes, in which a “synthetic lethality” approach could be promising [75]. According to this strategy, a synthetic lethal relationship is seen when a cancer specific gene mutation combined with the therapeutic targeting of another pathway results in the selective killing of mutated cancer cells without affecting the normal ones. The most exciting results of this approach are exemplified by the treatment with PARP inhibitors of *BRCA1-2* mutated ovarian cancers. Tumor cells carrying the mutations of the tumor suppressor genes *BRCA1* or *BRCA2* are defective in homologous recombination, and thus are sensitive to the inhibition of PARP activity, another key pathway involved in DNA repair [76, 77]. Combination of chemotherapy and targeted therapy or immunotherapy represents an alternative promising strategy in this setting.

Another important issue is the availability of tumor tissue for pathological and molecular studies during the course of the disease. Locally-advanced HNSCC could receive concurrent chemo-radiation therapy or induction therapy and patients without clinical complete response could undergo salvage surgery; residual neck disease may be present in as many as 30-60% of patients after the completion of chemo-radiation treatment [78–81]. As the neoadjuvant setting in breast cancer [82], this condition represents a suitable setting for evaluating new compounds because of the possibility to analyze tumor tissue before and after treatment as well as to obtain and compare anatomical and functional imaging.

In HNSCC treatment, another key point to consider is HPV status; currently, the HPV-status is associated with an improved outcome but it has no current relevance in terms of clinical management [83]. The most recent molecular platforms showed no significant difference in terms of frequency of mutations between HPV-negative and HPV-positive tumors, but a different molecular profile [37, 46]. These emerging data also showed a higher frequency of oncogene alterations and a lack of common tumor suppressor gene mutations in HPV-positive patients, making them more suitable for targeted therapy experimental approach. More broadly, molecular subtypes identified in both HNSCC and LSCC according to gene-expression data could be useful to design clinical studies with new compounds. Recently, in order to investigate individualized treatment for patients carrying different molecular subtypes of LSCC, Wu D. and colleagues evaluated the response to 24 drugs at eight dosages among 17 cell lines including all four LSCC subtypes. In this drug-response experiment, the authors demonstrated that the majority of cell lines responded to five of the 24 drugs (Panobinostat, 17-AAG, Irinotecan, Topotecan, and Paclitaxel) and the secretive subtype was significantly less sensitive to the drugs tested probably because of lower proliferation score than other subtypes [84]. Also in the latest molecular classification of HNSCC, the authors tested the hypothesis that each molecular subtype might have specific sensitivity to different drugs and they demonstrated a statistically significant difference for patients belonging to different subgroups [31].

### Immunotherapy

The chance to enhance immunological response of the host against tumors is at the basis of important advances in the treatment of solid tumors, from the successful story of immunotherapy in melanoma to the most recent success in NSCLC [85–87]. PD-1 is an immune checkpoint receptor expressed on activated T cells, dampening the immune response; PD-1 pathway blockade with monoclonal antibodies restores tumor-specific T cell-mediated immunity [88].

Recently the anti-PD-1 antibody nivolumab has been approved by Food and Drug Administration (FDA) and European Medicines Agency (EMA) for the treatment of previously treated metastatic LSCC according to the results of the phase III trial showing a significantly improvement in median overall survival: 9.2 months with nivolumab compared to 6.0 months with docetaxel (hazard ratio: 0.59; 95% CI: 0.44 to 0.79; *P* < 0.001), with prolonged persistence of clinical benefit in responders [89].

Molecular predictive markers of sensitivity to immunotherapy are still under investigation; in this trial nivolumab improved survival regardless of PD-L1 expression, while in non-squamous setting the expression of PDL-1, whatever cut-off used, demonstrated to be predictive of benefit from immunotherapy with respect to chemotherapy [90]. On the other hand, Herbst and colleagues observed that the responses to the anti-PD-L1 MPDL3280A were associated with high expression of PD-L1 especially by tumor-infiltrating immune cells [91]. Interestingly, most trials evaluating anti-PD-1 or anti-PD-L1 antibodies suggested that former and current smokers might preferentially benefit from immunotherapy [92]. This finding could be related to the higher overall mutational burden in these patients resulting in more tumor neoantigens and increased immunogenicity. Recently, whole-exome sequencing of NSCLC cases treated with the anti-PD1 pembrolizumab, revealed that higher non-synonymous mutation burden, higher neoantigen burden, and molecular smoking signature, characterized by high nucleotide transversion rate, molecular smoking signature (high nucleotide transversion rate) were associated with improved objective response, durable clinical benefit and progression-free survival [93]. Similarly, another study showed that tumors with genetic mismatch repair defects, which are associated with a high degree of mutational burden, respond better to PD-1 inhibitor compared with tumors proficient in mismatch repair. In this trial, clinical benefit was observed across tumors with mismatch repair deficiency, including cancer of the colon, stomach, uterus, duodenum, prostate, and bile ducts, providing compelling evidence that neoantigens in tumor with high mutational burden may represent a potential immune-target [94].

Similarly, several trials with immunotherapeutic agents are ongoing in HNSCC, as well as the analysis of predictive biomarkers, including HPV status [95]. To date, the most advanced results are from the KEYNOTE-012 expansion cohort study evaluating pembrolizumab in recurrent or metastatic HNSCC. The drug demonstrated a clinically meaningful overall response rate of 18.2% in this setting, in both HPV-positive and HPV-negative patients [96].

Molecular characterization according to the above discussed subtypes may also have a role in predicting the response to immunotherapy [27–31]; indeed, the molecular subtypes characterized by alterations in pathways involved in the immune response against cancer (Figure 2) could be suitable for new immunotherapeutic approaches. Supporting this idea, the inflamed phenotype signature, according to the classification by Keck *et al*., has shown to be a strong predictor of clinical benefit from anti-PD-1 treatment for HNSCC patients enrolled in KEYNOTE-012 study [96]. Moreover, therapeutic vaccines against HPV are under evaluation (NCT01493154) also in association with immunomodulatory therapies to increase sensitivity in poorly immunogenic tumors by restoring cytotoxic T cell activity [97].

## CONCLUSIONS

Molecular characterization of LSCC and HNSCC is one of the emerging burning issues in cancer translational research. On the basis of recent wide genetic characterization, the complexity and heterogeneity of genetic alterations in the two diseases represent a new fascinating biological model. The potential therapeutic implications stem from data of targeted therapy in other solid tumors, in particular lung adenocarcinomas, and from recent successful results of immunotherapy in LSCC. New molecularly-driven clinical trials of targeted therapy in selected subsets of patients, in parallel with the investigation about the association of conventional and targeted therapy or immunotherapy are warranted in order to test potential synergistic effects aiming to long-lasting disease control in advanced stage.
